# Laminar and Cellular Distribution of Monoamine Receptors in Rat Medial Prefrontal Cortex

**DOI:** 10.3389/fnana.2017.00087

**Published:** 2017-09-28

**Authors:** Noemí Santana, Francesc Artigas

**Affiliations:** ^1^Systems Neuropharmacology, Department of Neurochemistry and Neuropharmacology, Institut d’Investigacions Biomèdiques de Barcelona, Consejo Superior de Investigaciones Científicas, Barcelona, Spain; ^2^Centro de Investigación Biomédica en Red de Salud Mental, Madrid, Spain; ^3^Institut d’Investigacions Biomèdiques August Pi i Sunyer, Barcelona, Spain

**Keywords:** 5-hydroxytryptamine (serotonin) receptors, antidepressant drugs, antipsychotic drugs, cortical layers, dopamine receptors, major depressive disorder, noradrenaline receptors, schizophrenia

## Abstract

The prefrontal cortex (PFC) is deeply involved in higher brain functions, many of which are altered in psychiatric conditions. The PFC exerts a top-down control of most cortical and subcortical areas through descending pathways and is densely innervated by axons emerging from the brainstem monoamine cell groups, namely, the dorsal and median raphe nuclei (DR and MnR, respectively), the ventral tegmental area and the *locus coeruleus* (LC). In turn, the activity of these cell groups is tightly controlled by afferent pathways arising from layer V PFC pyramidal neurons. The reciprocal connectivity between PFC and monoamine cell groups is of interest to study the pathophysiology and treatment of severe psychiatric disorders, such as major depression and schizophrenia, inasmuch as antidepressant and antipsychotic drugs target monoamine receptors/transporters expressed in these areas. Here we review previous reports examining the presence of monoamine receptors in pyramidal and GABAergic neurons of the PFC using double *in situ* hybridization. Additionally, we present new data on the quantitative layer distribution (layers I, II–III, V, and VI) of monoamine receptor-expressing cells in the cingulate (Cg), prelimbic (PrL) and infralimbic (IL) subfields of the medial PFC (mPFC). The receptors examined include serotonin 5-HT_1A_, 5-HT_2A_, 5-HT_2C_, and 5-HT_3_, dopamine D_1_ and D_2_ receptors, and α_1A_-, α_1B_-, and α_1D_-adrenoceptors. With the exception of 5-HT_3_ receptors, selectively expressed by layers I–III GABA interneurons, the rest of monoamine receptors are widely expressed by pyramidal and GABAergic neurons in intermediate and deep layers of mPFC (5-HT_2C_ receptors are also expressed in layer I). This complex distribution suggests that monoamines may modulate the communications between PFC and cortical/subcortical areas through the activation of receptors expressed by neurons in intermediate (e.g., 5-HT_1A_, 5-HT_2A_, α_1D_-adrenoceptors, dopamine D_1_ receptors) and deep layers (e.g., 5-HT_1A_, 5-HT_2A_, α_1A_-adrenoceptors, dopamine D_2_ receptors), respectively. Overall, these data provide a detailed framework to better understand the role of monoamines in the processing of cognitive and emotional signals by the PFC. Likewise, they may be helpful to characterize brain circuits relevant for the therapeutic action of antidepressant and antipsychotic drugs and to improve their therapeutic action, overcoming the limitations of current drugs.

## Introduction

The prefrontal cortex (PFC) is the association cortex of the frontal lobe, located in its most rostral part. It has poorly defined anatomical boundaries although in all examined mammalian brains, it is defined by its connectivity with the mediodorsal nucleus of the thalamus. According to the original definition by Brodman, the human PFC contains areas 8–14 and 44–47, although other classifications also include ventromedial areas 14 and 25. The human PFC consists of three main regions: lateral, medial, and orbital. Orbital and ventromedial regions are mainly involved in emotional behavior whereas lateral areas (particularly the dorsolateral PFC) are involved in cognitive control. In the rat, the PFC contains four main regions, medial, lateral, ventral, and orbital, each containing several subdivisions that may vary according to different authors (Uylings et al., 2003; Dalley et al., 2004; Swanson, 2004; Paxinos and Watson, 2005; Fuster, 2008; Herculano-Houzel et al., 2013). See Fuster (2008) for extended information on PFC anatomy.

In primates, the PFC is dedicated to the representation, planning and execution of actions under a temporal pattern. It is involved in many higher brain functions, such as perception, attention, memory, language, intelligence, consciousness, affect, etc., and plays a key role in cognitive processes, such as working memory and executive functions (Miller, 2000; Fuster, 2001, 2008; Miller and Cohen, 2001). Automatic or stereotyped behaviors are bottom-up processes carried out by an innate connectivity between sensory and motor areas and do not require the engagement of the PFC (e.g., to look at a place where we hear a sudden noise). In contrast, the PFC involvement is required in situations with a large number of degrees of freedom, i.e., when flexibility is required to behave in a novel, unexpected or non-familiar environment (e.g., a EU or United States citizen driving in United Kingdom for the first time) or when behavioral rules change (Miller, 2000; Miller and Cohen, 2001; Buschman and Miller, 2007; Fuster, 2008). There is general consensus from multiple studies that the PFC reaches internally represented goals, and does this by coordinating sensory and motor processes of a lower association level. This process is thought to be influenced by the very large number of afferent and efferent connections to and from sensory and motor cortical and subcortical areas. As frequently summarized, a key feature of the PFC is a multi-layered architecture where sensory information is received from the external world, and emotional and contextual information is received and stored from limbic and temporal areas. The architecture further incorporates important intrinsic processing among the different subdivisions of the PFC itself. Projections to cortical premotor and motor areas and to the basal ganglia enable the performance of motor acts once a particular behavior has been selected (see, among other references, Bates and Goldman-Rakic, 1993; Lu et al., 1994; Jueptner et al., 1997a,b; Groenewegen and Uylings, 2000; Calzavara et al., 2007; Friedman et al., 2016). By virtue of this connectivity, the PFC can be considered at the highest level of the cortical areas, exerting a “top-down” control of behavior from a selection among multiple internally represented possible scenarios. A function specific to the PFC in cognitive control is the active maintenance of the neural activity that represents goals as well as the means to achieve these (see Miller, 2000 for further elaboration).

Working –or short-term- memory is a key function of the PFC. This capacity for sustained neuronal activity in the absence of sensory stimuli and even in the presence of distractors, allows the PFC to store and combine information for short periods of time before the execution of a given task. This property was discovered in the early 1970s by Fuster (1973) in primates and was subsequently reproduced and characterized by many groups (Funahashi et al., 1989; Miller et al., 1996; Romo et al., 1999; Arnsten, 2009). Interestingly, monoaminergic inputs to PFC (see below) play a crucial role in working memory capacity (Sawaguchi and Goldman-Rakic, 1991; Williams and Goldman-Rakic, 1995; Vijayraghavan et al., 2007). In particular, dopamine (DA) depletion in PFC induces cognitive deficits in monkeys similar to those evoked by removal of the frontal lobes (Brozoski et al., 1979).

### Prefrontal Cortex Connectivity

Broadly similar to other cortical areas, the PFC is composed of ∼75–80% glutamatergic pyramidal projection neurons, and ∼20–25% GABAergic local circuit interneurons (see Beaulieu, 1993 for an early report). The functions of the PFC rely closely on its connectivity with a vast array of other cerebral structures (Fuster, 2001). Excitatory glutamatergic afferent inputs originate from parts of the amygdala and hippocampus, from other cortical areas, and from a number of thalamic nuclei, including the mediodorsal, centromedial, and several midline nuclei. Pyramidal cell excitation is sculpted by inhibitory inputs, mainly from local GABAergic inputs. The multiple interneuron subtypes have been classified by anatomical and neurochemical features, and their selective targeting of pyramidal cell postsynaptic domains (Kawaguchi and Kondo, 2002; DeFelipe et al., 2013).

The PFC also receives a dense innervation from the brainstem monoaminergic nuclei: dorsal and median raphe nuclei, *locus coeruleus* and ventral tegmental (VTA) area, which employ serotonin (5-hydroxytryptamine, 5-HT), noradrenaline (NA) and dopamine (DA) as main neurotransmitters, respectively. These neuronal groups exert an important modulatory role of the excitatory and inhibitory currents in PFC neurons (Steinbusch, 1981; Van Eden et al., 1987; Aston-Jones and Cohen, 2005; Puig et al., 2005; Celada et al., 2013; Chandler et al., 2014) which are particularly relevant for the control of executive functions of PFC (Dalley et al., 2004; Robbins and Arnsten, 2009).

In turn, brainstem monoamine groups are innervated by descending axons from layer V pyramidal neurons in the medial PFC –mPFC- (for an overall view, see Gabbott et al., 2005) which control monoamine neuron activity (Thierry et al., 1979, 1983; Sara and Hervé-Minvielle, 1995; Hajós et al., 1998; Jodo et al., 1998; Celada et al., 2001; Martin-Ruiz et al., 2001), thus establishing a reciprocal connectivity and mutual control. These PFC-brainstem loops are relevant for the pathophysiology and treatment of psychiatric disorders, since (i) many psychiatric symptoms involve alterations of PFC functions, such as cognitive and emotional control, and (ii) psychiatric medications act either on presynaptic monoamine terminals (antidepressants blocking 5HT and/or NA transporters) or on postsynaptic monoamine receptors. Moreover, the ventral anterior cingulate cortex (vACC) has emerged as a key area in the pathophysiology and treatment of major depressive disorder (MDD), particularly in the mechanism of action of fast-acting antidepressant strategies such as deep brain stimulation (Mayberg et al., 2005; Puigdemont et al., 2011) and ketamine (Zarate et al., 2006). Hence, early neuroimaging studies reported on a reduced energy metabolism in the vACC (subgenual) of MDD patients. Further studies indicated an increased activity of the adjacent Brodmann area 25, which normalized after effective treatments, including deep brain stimulation. Likewise, optogenetic stimulation of the infralimbic cortex (IL, rodent equivalent of vACC) in rats mimicked the rapid and persistent antidepressant-like effects of systemic ketamine administration (Fuchikami et al., 2015) and the stimulation of AMPA receptors in IL (but not in the adjacent prelimbic cortex, PrL) evokes robust antidepressant-like effects, which involve an increased serotonergic activity and depend on an intact serotonergic system (Gasull-Camós et al., 2017).

Collectively, primate studies support a key role of dorsal and lateral PFC in cognition, and of ventromedial areas in the processing of emotional signals, although is still unclear whether equivalent areas in rodent PFC play similar roles. Given our interest in the pathophysiology and treatment of MDD and schizophrenia, we undertook a long-lasting effort to study the cellular and neurochemical elements involved in PFC-based circuits, in particular those existing between the PFC and brainstem monoamine nuclei. Here we summarize and review the histological data relative to the expression of the mRNAs encoding nine monoamine receptors (serotonin 5-HT_1A_-R, 5-HT_2A_-R, 5-HT_2C_-R and 5-HT_3_-R, dopamine D_1_-R and D_2_-R and α_1A_-, α_1B_-, and α_1D_-adrenoceptors) in pyramidal and GABAergic neurons of the mPFC, paying special attention to their layer distribution in the different subfields of the rat mPFC. Further studies will examine the expression of other relevant monoamine receptors, such as 5-HT_4_-R, 5-HT_6_-R, 5-HT_7_-R α_2_-adrenoceptors or β-adrenoceptors.

## Expression of Monoamine Receptors By PFC Neurons In Rat Brain

**FIGURE 1:**
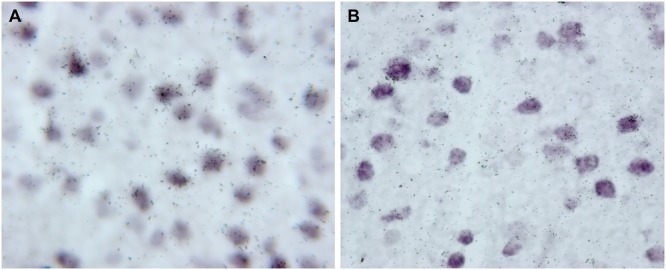
High magnification photomicrographs showing the presence of 5-HT_1A_ receptor mRNA (^33^P-labeled oligonucleotides) in pyramidal cells, identified by the presence of VGLUT1 mRNA (Dig-labeled oligonucleotides). Both images were acquired from the same experiment and correspond to deep layers of mPFC cingulate area. **(A)** Was captured in 2003 with a Nikon Eclipse E1000 microscope (Nikon, Tokyo, Japan) using a digital camera (DXM1200 3.0; Nikon) and analySIS Software (Soft Imaging System GmbH, Germany); **(B)** was captured in 2017 with a Zeiss Axioplan microscope equipped with a digital camera (XC50, Olympus) with Olympus CellSens Entry software.

The PFC contains a large number of pyramidal neurons and GABAergic interneurons expressing the mRNAs encoding the nine monoamine receptors examined, as reported elsewhere (Amargós-Bosch et al., 2004; Puig et al., 2004; Santana et al., 2004, 2009, 2013; Santana and Artigas, 2017). In all them we report on the cellular expression of the corresponding mRNAs in the different PFC subfields. However, in some studies we did not analyze the layer distribution of mRNAs. Therefore, in order to ensure data homogeneity and quality, we performed new cell counts on hybridized tissue sections corresponding to all previous studies (see Santana and Artigas, 2017 for analysis methodology), after checking that old and new cell counts were comparable. Remarkably, despite the long time spent since initial studies (e.g., the expression of 5-HT_1A_-R, 5-HT_2A_-R, and 5-HT_3_-R mRNA was examined in 2003–2004) old and new data are fully coincident, which indicates an excellent preservation of radioactive (silver grains) and non-radioactive (digoxigenin) signals, as show in **Figure 1**.

**FIGURE 2:**
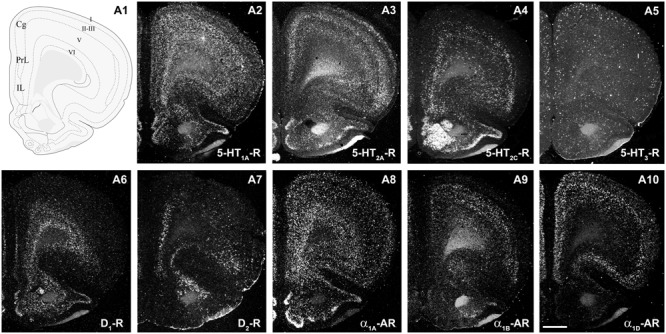
mRNA expression of monoaminergic receptors in rat prefrontal cortex (PFC). **(A1)** Coronal diagram from the rat brain atlas Swanson (2004) (used under CC BY-NC 4.0) at the approximate AP coordinate where cell counts have been performed in the cingulate (Cg), prelimbic (PrL), and infralimbic (IL) subdivisions. **(A2–A10)** Emulsion dipped dark-field PFC coronal sections hybridized with ^33^P-labeled oligonucleotide probes against the mRNAs encoding 5-HT_1A_-R **(A2)**, 5-HT_2A_-R **(A3)**, 5-HT_2C_-R **(A4)**, 5-HT_3_-R **(A5)** dopamine D_1_-R **(A6)**, dopamine D_2_-R **(A7)** and α_1A_-, α_1B_- and α_1D_-adrenoceptors (**A8–A10**, respectively). Bar: 1 mm. See Puig et al. (2004), Santana et al. (2004, 2009, 2013), Santana and Artigas (2017) for detailed methods.

**FIGURE 3:**
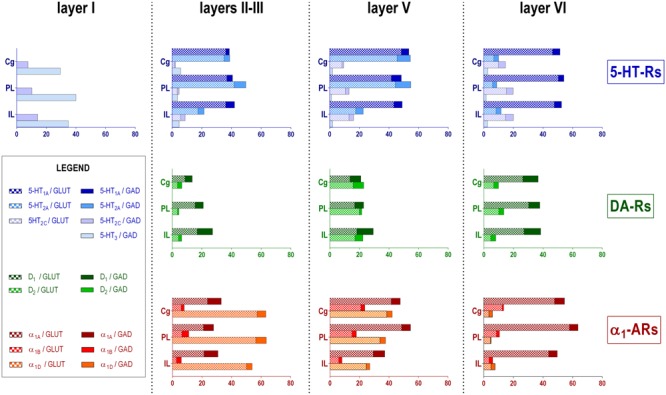
Bar graphs showing the expression of each monoamine receptor mRNA in the different layers and PFC subfields of the mPFC. Data (mean of 3 rats, 2–3 consecutive sections per rat) are the percentages of pyramidal (VGLUT1-positive) or GABAergic (GAD-positive) neurons expressing each mRNA, expressed as percentages of the total neuronal population, assuming a standard 80% of pyramidal neurons and 20% of GABAergic interneurons. As layer I lacks glutamatergic neurons, layer I data is expressed as percentages of GAD-positive cells containing each receptor mRNA. Cg, anterior cingulate cortex; PL, prelimbic cortex; IL infralimbic cortex. Layer I, II–III, V, and VI stand for medial prefrontal cortical layers I to VI, respectively.

**Figure 2** shows the localization of the mRNAs encoding serotonergic receptors (5-HT_1A_-R, 5-HT_2A_-R, 5-HT_2C_-R, and 5-HT_3_-R), dopamine D_1_-R and D_2_-R and α_1A_-, α_1B_- and α_1D_-adrenoceptors in coronal sections of rat PFC. **Table 1** shows the percentages of pyramidal neurons (vGLUT1-positive) and GABAergic interneurons (GAD-positive) expressing each of the 9 receptors in the different mPFC subfields (cingulate –Cg-, prelimbic –PrL-, and infralimbic –IL-) and PFC layers. **Figure 3** shows the same data, expressed as percentages of the *total neuronal population*, assuming a standard 80% of pyramidal neurons and 20% of GABAergic interneurons.

**Table 1:** Percentages of pyramidal and GABAergic neurons expressing monoamine receptor mRNAs in rat mPFC.

		VGLUT1			GAD			
		Layer II–III	Layer V	Layer VI	Layer I	Layer II–III	Layer V	Layer VI
	Cg	45.1 ± 2.8	60.3 ± 0.9	58.0 ± 2.7		12.5 ± 1.0	25.7 ± 2.0	25.5 ± 0.3
**5-HT_1A_-R**	PrL	46.0 ± 3.2	52.0 ± 4.5	62.9 ± 4.3		19.0 ± 2.9	33.7 ± 0.7	19.0 ± 0.6
	IL	45.1 ± 2.7	54.0 ± 6.7	59.5 ± 2.4		29.5 ± 4.3	28.2 ± 2.8	24.7 ± 1.8
	Cg	43.8 ± 2.7	56.8 ± 1.2	8.5 ± 1.7		18.8 ± 3.9	44.8 ± 6.4	16.1 ± 4.7
**5-HT_2A_-R**	PrL	52.2 ± 4.4	55.3 ± 1.4	7.5 ± 0.6		39.5 ± 1.1	52.2 ± 4.8	14.0 ± 3.9
	IL	21.8 ± 4.4	22.0 ± 5.3	10.3 ± 1.6		20.4 ± 5.2	25.1 ± 7.0	17.1 ± 6.0
	Cg	1.7 ± 0.4	10.3 ± 1.8	12.3 ± 1.5	7.6 ± 3.3	4.6 ± 1.5	5.7 ± 1.7	23.9 ± 2.9
**5-HT_2C_-R**	PrL	4.3 ± 2.2	13.2 ± 3.6	19.2 ± 2.2	10.3 ± 2.7	6.6 ± 1.2	12.4 ± 2.1	22.5 ± 2.7
	IL	7.3 ± 1.0	16.2 ± 1.3	18.3 ± 0.8	14.1 ± 2.4	14.8 ± 3.9	15.7 ± 3.9	26.9 ± 6.1
	Cg				29.5 ± 6.1	28.7 ± 3.6	9.1 ± 2.0	13.3 ± 3.7
**5-HT_3_-R**	PrL				40.0 ± 2.1	18.3 ± 1.5	5.7 ± 0.9	8.0 ± 0.6
	IL				34.9 ± 7.4	23.9 ± 2.1	9.5 ± 0.8	12.4 ± 1.5
	Cg	10.8 ± 0.1	16.9 ± 1.0	32.9 ± 5.9		24.7 ± 1.1	36.8 ± 8.0	51.9 ± 4.2
**D_1_-R**	PrL	19.2 ± 3.2	20.9 ± 1.5	37.9 ± 3.2		28.1 ± 1.0	30.5 ± 1.6	37.5 ± 3.6
	IL	21.0 ± 0.4	22.8 ± 0.8	33.8 ± 2.6		52.0 ± 3.3	55.5 ± 6.2	56.8 ± 2.0
	Cg	4.5 ± 1.8	19.5 ± 0.4	8.3 ± 1.7		14.5 ± 5.3	36.4 ± 6.2	16.2 ± 1.4
**D_2_-R**	PrL	4.5 ± 1.0	24.9 ± 1.6	12.5 ± 0.5		4.7 ± 2.2	7.9 ± 2.3	17.2 ± 1.2
	IL	5.3 ± 1.1	21.1 ± 2.1	5.8 ± 0.4		10.9 ± 2.9	27.1 ± 2.3	17.2 ± 3.2
	Cg	29.6 ± 3.7	51.9 ± 10.7	59.7 ± 3.0		46.7 ± 7.2	30.4 ± 1.0	34.1 ± 10.9
**Alpha_1A_-AR**	PrL	26.2 ± 3.5	60.7 ± 6.2	72.3 ± 4.6		34.4 ± 4.8	30.4 ± 1.5	28.8 ± 4.2
	IL	26.7 ± 5.7	36.6 ± 3.1	54.7 ± 2.9		47.4 ± 2.8	39.3 ± 5.5	29.4 ± 1.5
	Cg	7.4 ± 1.6	26.2 ± 0.0	15.5 ± 2.1		10.4 ± 3.5	13.5 ± 2.4	5.2 ± 2.9
**Alpha_1B_-AR**	PrL	7.9 ± 2.1	18.7 ± 3.5	10.7 ± 1.4		23.7 ± 4.8	14.7 ± 3.5	10.3 ± 2.1
	IL	3.3 ± 0.6	7.3 ± 1.5	4.5 ± 1.0		16.8 ± 1.6	11.3 ± 5.6	12.5 ± 1.4
	Cg	71.6 ± 5.6	48.2 ± 2.1	4.4 ± 1.5		29.8 ± 3.8	18.4 ± 2.4	11.9 ± 3.6
**Alpha_1D_-AR**	PrL	70.5 ± 3.5	42.3 ± 1.4	5.7 ± 2.6		35.6 ± 4.0	19.0 ± 9.4	2.1 ± 1.2
	IL	62.7 ± 2.6	30.8 ± 0.9	6.1 ± 1.4		19.1 ± 3.7	11.9 ± 6.1	13.8 ± 9.0

*Data are means ± standard errors of the mean of 3 rats (2–3 adjacent sections per rat) and show the percentage of pyramidal (VGLUT1-positive) or GABAergic (GAD-positive) neurons expressing each monoamine receptor mRNA in layers I, II–III, V, and VI of the cingulate (Cg), prelimbic (PrL), and infralimbic (IL) subdivisions of the mPFC.*

**FIGURE 4:**
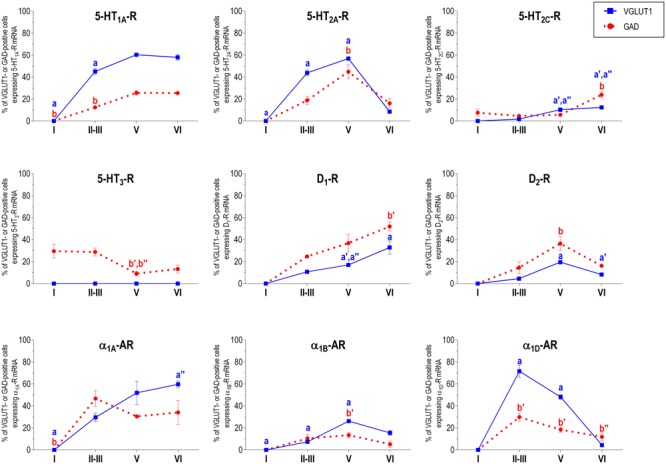
Percentages of pyramidal (VGLUT1-positive) and GABA (GAD-positive) neurons expressing the nine monoamine receptors studied in the different layers of cingulate area of medial prefrontal cortex. ^a^*p* < 0.05 vs. rest of layers, ^a^′*p* < 0.05 vs. layer I; ^a^″′*p* < 0.05 vs. layer II–III; ^a^″′*p* < 0.05 vs. layer V (for VGLUT1 graphs). ^b^*p* < 0.05 vs. rest of layers, ^b^′*p* < 0.05 vs. layer I; ^b″^*p* < 0.05 vs. layers II–III; ^b^″′*p* < 0.05 vs. layer V (for GAD graphs), one-way ANOVA followed by Tukey’s test.

**FIGURE 5:**
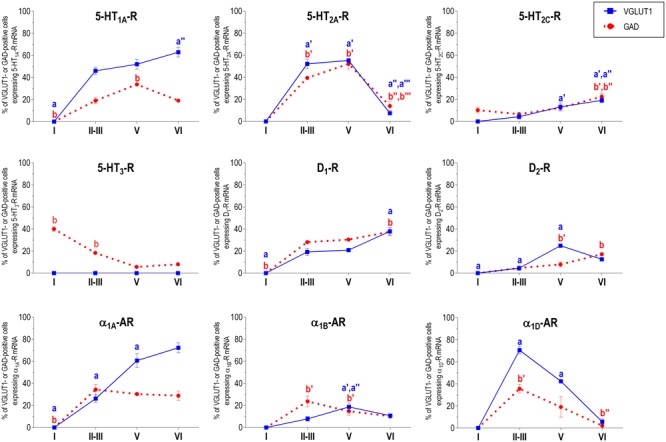
Percentages of pyramidal (VGLUT1-positive) and GABA (GAD-positive) neurons expressing the nine monoamine receptors studied in the different layers of prelimbic area of medial prefrontal cortex. ^a^*p* < 0.05 vs. rest of layers, ^a^′*p* < 0.05 vs. layer I; ^a″^*p* < 0.05 vs. layers II–III; ^a^″′*p* < 0.05 vs. layer V (for VGLUT1 graphs). ^b^*p* < 0.05 vs. rest of layers, ^b^′*p* < 0.05 vs. layer I; ^b″^p < 0.05 vs. layers II–III; ^b^″′*p* < 0.05 vs. layer V (for GAD graphs), one-way ANOVA followed by Tukey’s test.

**FIGURE 6:**
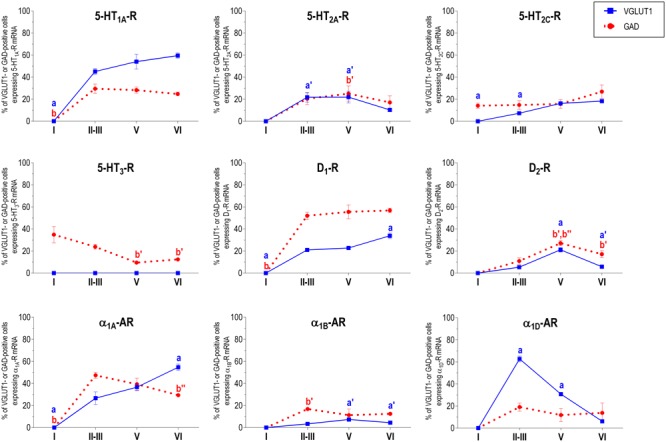
Percentages of pyramidal (VGLUT1-positive) and GABA (GAD-positive) neurons expressing the nine monoamine receptors studied in the different layers of infralimbic area of medial prefrontal cortex. ^a^*p* < 0.05 vs. rest of layers, ^a^′*p* < 0.05 vs. layer I; ^a″^*p* < 0.05 vs. layers II–III; ^a^″′*p* < 0.05 vs. layer V (for VGLUT1 graphs). ^b^*p* < 0.05 vs. rest of layers, ^b^′*p* < 0.05 vs. layer I; ^b″^*p* < 0.05 vs. layers II–III; ^b^″′*p* < 0.05 vs. layer V (for GAD graphs), one-way ANOVA followed by Tukey’s test.

With the exception of 5-HT_3_-R, exclusively expressed in GABAergic interneurons located mainly in superficial layers I-III, the rest of monoaminergic receptors are present in both neuronal types in varying proportions, and in middle (II–III) and deep (V–VI) layers of the Cg, PrL, and IL subfields. **Figures 4–6** show the percentages of pyramidal and GABAergic neurons expressing each receptor across layers in the three mPFC subfields (Cg, PrL, and IL, respectively).

Some receptors are highly co-localized (5-HT_1A_-R and 5-HT_2A_-R, Amargós-Bosch et al., 2004; 5-HT_2A_-R and α_1_-adrenoceptors, Santana et al., 2013) while others show little overlap (D_1_-R and D_2_-R, Santana et al., 2009). This distribution suggests a complex monoaminergic control of PFC activity, with some convergent actions on certain neuronal populations together with selective actions on other neuronal populations. In the following sections, we summarize the most important features of receptor expression in pyramidal and GABAergic neurons of the different PFC layers.

### Layer I

Only two of the nine receptors examined (5-HT_2C_-R and 5-HT_3_-R) were expressed by GABA interneurons of layer I. There is a greater proportion of GABA interneurons expressing 5-HT_3_-R (30–40%) than 5-HT_2C_-R (8–14%; values in parentheses refer to the range of values in the three mPFC subfields: **Table 1**).

**FIGURE 7:**
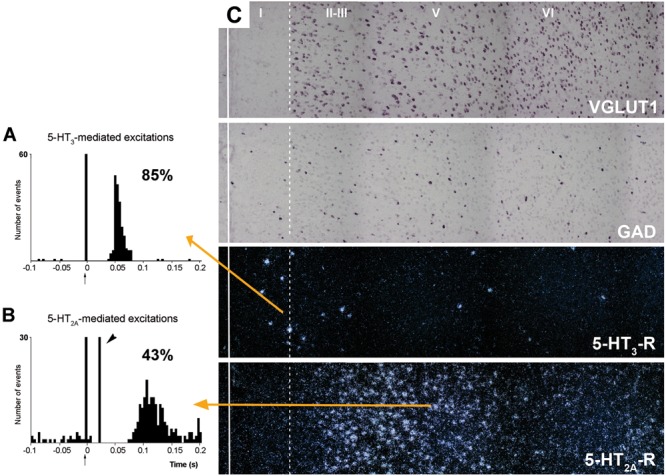
**(A,B)** are peristimulus time histograms showing the orthodromic excitations elicited by the electrical stimulation of the DR at a physiological rate (0.5–1.7 mA, 0.2 ms square pulses, 0.9 Hz) on **(A)** a putatively GABAergic, 5-HT_3_-R-expressing neuron and **(B)** on a layer V pyramidal neuron in the prelimbic PFC, identified by antidromic stimulation from midbrain (note the antidromic potential, arrowhead). Both responses were selectively blocked by the administration of the respective antagonists ondansetron **(A)** and M100907 **(B)** (not shown). The 5-HT_3_-R-mediated responses in putative GABAergic neurons were faster and more effective than those evoked by 5-HT_2A_-R activation in pyramidal neurons, due to the ionic nature of 5-HT_3_-R. The concordance rates of the units shown are 85% **(A)** and 43% **(B)**, i.e., 100 electric stimuli delivered in the DR evoked 80 action potentials in GABAergic interneurons through 5-HT_3_-R activation, compared to 45 action potentials evoked in layer V pyramidal neurons, mediated by the activation of 5-HT_2A_-R. Each peristimulus consists of 200 triggers; bin size is 4 ms. The arrow at zero abscissa marks the stimulation artifact. **(C)** Composite photomicrographs showing the localization of cells expressing VGLUT1, GAD, 5-HT_3_-R, and 5-HT_2A_–R mRNAs through layers I–VI at the level of the prelimbic PFC. The continuous vertical line denotes the location of the midline whereas the dotted line shows the approximate border between layers I and II. Pyramidal neurons (as visualized by VGLUT1 mRNA) are present in layers II–VI whereas GAD mRNA-positive cells are present in all layers, including layer I. Note the different location of cells expressing 5-HT_3_-R and 5-HT_2A_–R. 5-HT_3_-R transcript is expressed by a limited number of GABA interneurons in layers I–III, particularly in the border between layers I and II. However, they represent 40% of GABAergic neurons in layer I. Scale bar = 150 μm. Redrawn from Puig et al. (2004), by permission of Oxford University Press.

5-HT_2C_-Rs are G-protein coupled metabotropic receptors that activate the phospholipase C signaling pathway. It undergoes RNA editing, which dynamically regulates its constitutive activity, unique among 5-HT receptors (Berg et al., 2008; Werry et al., 2008; Aloyo et al., 2009; O’Neil and Emeson, 2012). On the other hand, the 5-HT_3_-R is the only ionotropic monoamine receptor. It is selectively expressed by a subpopulation of GABA interneurons not expressing parvalbumin or somatostatin, and displays strong actions on neuronal activity (Puig et al., 2004; Varga et al., 2009; Lee et al., 2010). **Figure 7** shows the rapid and robust excitatory action of endogenous 5-HT on layers I–III GABA neurons expressing 5-HT_3_-R and its comparison with the slower and more moderate activation of layer V pyramidal neurons by metabotropic 5-HT_2A_-R.

Despite its localization in upper cortical layers, GABA cells expressing 5-HT_3_-R tightly control the activity of pyramidal neurons located in deep layers. Hence, blockade of 5-HT_3_-R in rat brain by the selective antagonist ondansetron or by the new antidepressant drug vortioxetine (combining 5-HT transporter inhibition with 5-HT_3_-R blockade; Sanchez et al., 2015) markedly enhanced the discharge rate of layer V pyramidal neurons, identified by antidromic activation from midbrain (DR or VTA; Riga et al., 2016). Interestingly, ∼70% or the pyramidal neurons recorded were sensitive to 5-HT_3_-R blockade, a very high percentage taking into account the relative long distance between the cell bodies of both neuronal types. This action likely involves the attenuation of tonic layers I–III GABA inputs on the tufts of layer V pyramidal neurons, thus allowing excitatory inputs (possibly thalamocortical matrix inputs reaching layer I -Jones (2001))- to enhance pyramidal neuron activity.

Much less is known on the role played by layer I 5-HT_2C_-R on PFC neuronal activity. Unlike 5-HT_1A_-R and 5-HT_2A_-R, which exert a large variety of actions, including control the activity of pyramidal neurons and fast-spiking interneurons as well as cortical oscillations in rat mPFC (Araneda and Andrade, 1991; Amargós-Bosch et al., 2004; Puig et al., 2005, 2010; Celada et al., 2013), 5-HT_2C_-R are not involved in the latter effects. However, the relevance of 5-HT_2C_-R for cognitive and affective processes (Heisler et al., 2007; Boulougouris and Robbins, 2010; Pennanen et al., 2013) suggests its participation in the modulation of PFC-based circuits. Yet it is unclear whether the small receptor subpopulation in layer I GABAergic cells plays a significant role given its larger abundance of 5-HT_2C_-R in other PFC areas (**Figure 2**).

### Layers II–III

Unlike in layer I, supragranular layers II and III show a large abundance of serotonergic, dopaminergic and α_1_-adrenergic receptors, expressed by pyramidal and GABAergic neurons in all mPFC subfields (**Table 1** and **Figures 3–6**).

Most abundant 5-HT receptors in layers II–III are 5-HT_1A_-R and 5-HT_2A_-R, expressed by 45–52% of the pyramidal neurons and by 12–39% of GABA interneurons in Cg and PrL subfields, where they are abundantly co-expressed (Amargós-Bosch et al., 2004). Interestingly, the proportion of pyramidal neurons expressing 5-HT_2A_-R is lesser than that that expressing 5-HT_1A_-R in IL (45% vs. 22%, compared with 46–52% in PrL and 45–44% in Cg; see **Figures 4–6**). This suggests a predominance of inhibitory actions of 5-HT in the IL subfield. The differential excitation/inhibition balance in IL vs. PrL may be relevant to clarify the role of ventral cingulate areas in the pathophysiology and treatment of MDD, as summarized in the introduction. This difference is also common to layer VI, where a greater proportion of cells express 5-HT_1A_-R vs. 5-HT_2A_-R (**Table 1** and **Figures 4–6**), indicating a preferential inhibitory action of 5-HT on both intracortical and cortico-subcortical pathways arising from IL.

The proportion of 5-HT_3_-R-expressing GABAergic cells in layers II–III is lower than in layer I, reaching 18–29% in Cg, PrL, and IL. Similarly, there is a very low percentage of pyramidal (2–7%) and GABAergic neurons (5–15%) expressing 5-HT_2C_-R.

Dopamine modulates PFC function by multiple mechanisms, (Chen et al., 2004; Seamans and Yang, 2004) consistent with the presence of DA receptors in pyramidal neurons and GABAergic interneurons (**Table 1** and **Figures 4–6**). However, DA receptor expression by layers II–III cells was lower than for 5-HT and NA receptors, suggesting a comparatively less relevant role of DA in the tuning of the intracortical PFC output. The proportion of pyramidal cells expressing dopamine D_1_-R was lower than that expressed by GABA cells (11–21% vs. 25–52%, respectively), suggesting a predominantly inhibitory role of DA on the cortical PFC output via D_1_-R activation on GABAergic interneurons. This inhibitory action may be particularly relevant in IL, with a very large contribution of D_1_-R expressed in GABA interneurons. DA D_2_-R were expressed by an even lesser proportion of pyramidal and GABAergic neurons (4–5% vs. 5–15%, respectively).

Unlike 5-HT and α_1_-adrenoceptors, mainly expressed by a greater proportion of pyramidal neurons (except 5-HT_3_-R, selectively expressed by GABA interneurons), DA D_1_-R are expressed by a comparable or greater proportion of GABAergic than of pyramidal neurons. This expression pattern may be relevant to understand the inverted U relationship between D_1_-R activation and working memory performance (Williams and Goldman-Rakic, 1995). Hence, the activation of D_1_-R in GABAergic interneurons by an excess of endogenous DA (such as that produced by stress) may attenuate or cancel persistent neuronal activity evoked by D_1_-R activation in pyramidal neurons.

In contrast to DA receptors, the three α_1_-adrenoceptors were abundantly expressed in layers II–III, with α_1D_-adrenoceptors being expressed by more than 50% of PFC neurons in all PFC subfields (>60% in Cg and PrL), and with a similar ratio of pyramidal/GABA neurons in CG and PrL (lower proportion in IL GABA interneurons). The receptor expressed in the smaller neuronal proportion was the α_1B_-adrenoceptor (3–8% in pyramidal neurons, 10–24% in GABA interneurons).

Interestingly, α_1_-adrenocptors were co-expressed with 5-HT_2A_-R in varying proportions, depending on the receptor type and the mPFC subfield, with α_1A_- and α_1D_-adrenocepetors reaching a 80% co-expression in Cg (Santana et al., 2013). Although we did not perform a detailed layer analysis of co-expressing cells, the fields examined in the original study correspond mainly to layers II–III, with some contribution of deep layers in PrL and IL (Santana et al., 2013). Given the high co-expression of 5-HT_1A_-R and 5-HT_2A_-R mRNAs, and that of 5-HT_2A_-R with α_1_-adrenoceptors, it is likely that a substantial proportion of mPFC neurons express the three receptors. 5-HT_1A_-R and 5-HT_2A_-R are likely located in different cellular compartments and regulate different processes. Hence, 5-HT_2A_-R are possibly located in dendritic spines and modulate synaptic inputs (Marek and Aghajanian, 1999) whereas 5-HT_1A_-R in the axon hillock may regulate action potential generation, in a way similar to GABA_A_-R (DeFelipe et al., 2001). Indeed, excitatory and inhibitory responses have been recorded in the same pyramidal neurons after DR stimulation, supporting that both receptors are functionally relevant in the control of pyramidal neuron activity (Amargós-Bosch et al., 2004). However, despite 5-HT_2A_-R and α_1_-adrenoceptors share signaling pathways (G_q/11_ protein; Claro et al., 1993; Bartrup and Newberry, 1994; Berg et al., 1998) and there is evidence of heteromerization in artificial systems (Santana et al., unpublished observations), there is no evidence of an *in vivo* interaction between both receptors as yet.

In summary, layers II/III contain a very large proportion of pyramidal and GABAergic cells expressing 5-HT, α_1_-adrenoceptors, and –to a lesser extent- DA D_1_-R and D_2_-R, an observation indicating a crucial role of monoamines in the modulation of the connectivity between PFC and other cortical areas, as well as with subcortical structures also innervated by layers II–III pyramidal neurons, such as the basolateral amygdala, dorsal and ventral striatum and lateral hypothalamus (Gabbott et al., 2005).

### Layer V

Layer V contains the highest proportion of pyramidal and GABA neurons expressing monoamine receptors (**Table 1** and **Figures 4–6**). The proportion of pyramidal and GABA neurons expressing 5-HT receptors in layer V was very similar to that in layers II/III, yet with a greater abundance of glutamatergic cells expressing 5-HT_2C_-R (10–16% in layer V vs. 2–7% in the different subfields of layers II–III). 5-HT_1A_-R and 5-HT_2A_-R were expressed by 52–60% of pyramidal neurons (except in IL, just a 22%) and 25–52% of GABA interneurons. In contrast, the 5-HT_3_-R is expressed by only 6–10% of GABA neurons in this layer.

The expression of DA D_1_-R was similar to that in layers II–III, whereas a substantially greater proportion of layer V pyramidal neurons express DA D_2_-R (20–25% in layer V vs. 4–5% in layers II–III). The presence of both DA receptors in layer V neurons is consistent with previous electrophysiological data showing direct and GABA-mediated effects on layer V pyramidal neurons (Seamans and Yang, 2004; Tseng and O’Donnell, 2007). As discussed above for layers II/III the presence of a comparable or higher proportion of GABAergic interneurons than of pyramidal neurons may be related to the inverted U relationship between DA D_1_-R occupancy and working memory performance.

With regard to α_1_-adrenoceptors, there was a more balanced expression than in layers II–III, with similar or greater proportions of pyramidal neurons expressing α_1A_- vs. α_1D_-adrenoceptors and a greater proportion of neurons expressing α_1B_-adrenoceptors than in layers II–III, and with a marked dorso-ventral negative gradient in mPFC (**Table 1** and **Figures 4–6**). The presence of α_1_-adrenoceptors in layer V pyramidal and GABAergic neurons is consistent with previous electrophysiological reports showing that α_1_-adrenoceptor stimulation can elicit excitatory or inhibitory postsynaptic currents in layer V pyramidal neurons (Marek and Aghajanian, 1999; Luo et al., 2015). Interestingly, the excitatory postsynaptic currents evoked by 5-HT through 5-HT_2A_-R were several-fold greater than those evoked by NA and DA (Marek and Aghajanian, 1999), an effect perhaps related to the facilitation of intrinsic PFC networks by 5-HT, acting on subpopulation of pyramidal neurons strongly excited by 5-HT_2A_-R (Beique et al., 2007).

Given the large number of subcortical structures innervated by layer V pyramidal neurons (Gabbott et al., 2005), the wealth of monoamine receptors in this layer suggests a wide control of subcortical activity, including that of brainstem monoamine nuclei. Interestingly, layer V pyramidal neurons projecting to DR and/or VTA are highly sensitive to psychotomimetic drugs used as pharmacological models of schizophrenia, such as non-competitive NMDA-R antagonists and serotonergic hallucinogens. Remarkably, these actions on layer V pyramidal neurons are counteracted by antipsychotic drugs acting on DA and 5-HT receptors (Puig et al., 2003; Bortolozzi et al., 2005, 2007; Díaz-Mataix et al., 2006; Kargieman et al., 2007; Riga et al., 2014) suggesting a correlate of these drug actions with their therapeutic effect. Likewise, the fast antidepressant actions of ketamine are associated to an activation of layer V pyramidal neurons in the mPFC (Li et al., 2010).

### Layer VI

Most pyramidal neurons in layer VI of the PFC project to the mediodorsal nucleus of the thalamus (MD), whereas a smaller proportion project to dorsal and ventral striatum and to the lateral hypothalamus (Gabbott et al., 2005). In turn, MD fibers reach layers III–V of the PFC (Kuroda et al., 1998), thus establishing a reciprocal cortico-thalamocortical connectivity and mutual control. Additionally, PFC axons projecting to MD branch to innervate fast-spiking GABA neurons in the thalamic reticular nucleus, which provides feed-forward inhibition to excitatory thalamic nuclei, including MD (Pinault, 2004). The presence of an abundant population of layer VI pyramidal and GABAergic neurons expressing monoamine receptors indicates that the activity of thalamocortical networks is also modulated by monoamines.

Although layers V and VI are typically considered as “deep layers” and some electrophysiological studies assessing monoamine actions do not discriminate between them, there are substantial differences in the proportions of neurons expressing monoamine receptors (**Figures 4–6**), which supports different actions of the respective monoamines on both layers. Hence, while 5-HT_1A_-R are also expressed by a large proportion of pyramidal neurons (58–63% in layer VI vs. 52–60% in layer V), 5-HT_2A_-R were expressed by only 7–10% pyramidal neurons in layer VI, suggesting a predominantly inhibitory role of 5-HT on corticothalamic pathways. In contrast, the proportion of cells expressing 5-HT_2C_-R was greater than in layer V and greater than that expressing 5-HT_2A_-R.

Likewise, a remarkable difference exists in regards to catecholamine receptors, with a greater percentage of pyramidal neuros expressing DA D_1_-R than in layer V (33–38% vs. 17–23% in layer V) and a much lesser percentage of those expressing α_1D_-adrenoceptors (4–6% in layer VI vs. 31–48% in layer V) (**Figures 3–6**). Likewise, the proportion of pyramidal neurons expressing α_1B_-adrenoceptors was lower than in layer V and exhibited a marked negative DV gradient (15% in Cg, 11% in PrL, 4% in IL).

Collectively, these data indicates that cortico-thalamic pathways are strongly modulated by 5-HT_1A_-R, DA D_1_-R and α_1A_-adrenoceptors.

## Concluding Remarks

The PFC exerts a top-down control of brain activity thanks to its ample and reciprocal connectivity with cortical and subcortical brain structures, with the exception of the basal ganglia, which are connected with the PFC via thalamic nuclei (Groenewegen and Uylings, 2000; Miller and Cohen, 2001; Gabbott et al., 2005). Monoamine receptors in the various PFC layers and subfields are located in a key position to modulate the processing of cognitive and emotional signals by the PFC in physiological conditions (Robbins and Arnsten, 2009). In addition, antidepressant and antipsychotic drugs interact with most monoamine receptors in PFC, a process likely contributing to their therapeutic effects (Artigas, 2010, 2013). These actions involve (i) direct agonist/antagonist effects, as in the case of classical antipsychotic drugs blocking DA D_2_-R and D_1_-R, or second generation antipsychotic drugs, also targeting 5-HT_1A_-R, 5-HT_2A_-R, 5-HT_2C_-R and α_1_-adrenoceptors, or (ii) indirect agonist actions, derived from the blockade of 5-HT and/or NA transporters by antidepressant drugs. Additionally, some antidepressant drugs block monoamine receptors, such as trazodone, mirtazapine, agomelatine, or vortioxetine. The presence of monoamine receptors in all cortical layers indicates that psychoactive drugs control information processing in PFC-based circuits in a complex manner, through the modulation of excitatory inputs onto PFC pyramidal neurons, the control of local microcircuits via receptors located in GABA interneurons, and finally, through the modulation of the pyramidal output to subcortical structures. As an example of this complexity, 5-HT_3_-R blockade in layers I–III GABA cells (likely controlling thalamic inputs) enhances the discharge rate of layer V pyramidal neurons projecting to DR and/or VTA (Riga et al., 2016). In other words, 5-HT_3_-R located in a relatively small interneuron population modulates the interplay between the thalamic matrix, the PFC and brainstem monoamine cell groups.

Important, currently missing, information for better understanding monoamine function in the PFC would be to define the projection fields of pyramidal neurons expressing one or more receptors (Vázquez-Borsetti et al., 2009; Mocci et al., 2014). This information is relevant for understanding distal actions of drugs targeting monoamine PFC receptors, since an action on PFC receptors may immediately translate into neuronal activity changes in cortical and subcortical structures receiving PFC inputs. It is hoped that novel histological and tracing technologies will help to delineate the precise role of each monoamine receptor in the control of neuronal activity in cortical and subcortical areas, thus improving our understanding of the role of monoamines in PFC function.

## Author Contributions

FA and NS have planned and designed experiments, analyzed data and write the manuscript. NS performed experiments.

## Conflict of Interest Statement

The authors declare that the research was conducted in the absence of any commercial or financial relationships that could be construed as a potential conflict of interest.
